# Sentinel lymph node biopsy in early oral cavity tumors: Evaluation of the oncologic efficacy compared to elective neck dissection^☆^^☆☆^

**DOI:** 10.1016/j.bjorl.2025.101639

**Published:** 2025-06-04

**Authors:** Marco Roberto Seferin, Fábio Roberto Pinto, Chin Shien Lin, Ana Kober Nogueira Leite, Paulo Vitor Sola Gimenes, Rogerio Aparecido Dedivitis, Marco Aurélio Vamondes Kulcsar, Claudio Roberto Cernea, Leandro Luongo Matos

**Affiliations:** a Universidade de São Paulo (USP), Faculdade de Medicina (FM), Programa de Pós-Graduação, São Paulo, SP, Brazil; b Universidade de São Paulo (USP), Faculdade de Medicina (FM), Departamento de Cabeça e Pescoço, São Paulo, SP, Brazil; c Universidade de São Paulo (USP), Faculdade de Medicina (FM), Instituto do Câncer do Estado de São Paulo (ICESP), Departamento de Cabeça e Pescoço, São Paulo, SP, Brazil; d Universidade de São Paulo (USP), Faculdade de Medicina (FM), Hospital das Clínicas (HC), São Paulo, SP, Brazil

**Keywords:** Mouth neoplasms, Sentinel lymph node biopsy, Lymphatic metastasis, Prognosis, Survival

## Abstract

**Objective:**

To describe the oncological results of a prospective study in the use of sentinel lymph node biopsy as part of the surgical treatment of squamous cell carcinoma T1/T2N0 of the oral cavity in comparison to the results of patients submitted to levels I, II and III SND.

**Methods:**

It was a prospective study in which seventy patients were divided into two groups, 35 being submitted to SND and the other 35 to SLB.

**Results:**

In the SND group, locoregional recurrence occurred in 17.1%, with a 2.9% development of distant metastasis and 8.8% evolved with a second primary tumor. In the SLB, locoregional recurrence was observed in 7 patients (20.0%), 5.7% developed distant metastasis, and 5.7% had a second primary tumor. There was no significant difference between the two groups for both overall (*p* = 0.521) and disease-free survival (*p* = 0.753).

**Conclusion:**

A SLB is reliable for the management of clinically negative neck in patients with oral T1/T2N0 squamous cell carcinoma.

**Level of evidence:**

Level III.

## Highlights

• Locorregional recurrence did not differed between both groups.
• Overall survival between sentinel node biopsy and selective neck dissection group where not different.
• Disease free survival between sentinel node biopsy and selective neck dissection group where not different.

## Introduction

The high risk of metastasis for the regional lymph nodes is a highlighted characteristic of the malignant neoplasms affecting the upper digestive tract. This capacity results from the association between the aggressive biological behavior of a tumor to dissemination, potentiated by a typical lymphatic circulation in the anatomical location.^1^, ^2^ Even for patients who have the small primary tumor (T1, T2) and the clinically negative neck (cN0), the incidence of occult metastases may range from 10% to 50%.^3^, ^4^, ^5^ The presence of cervical metastatic lesions is considered the main prognostic factor for patients with squamous cell carcinomas of the head and neck.^6^, ^7^ Its presence is related to the decrease in survival by half in five years, as well as the increased risk of locally recurrent regional and distant metastases.^8^, ^9^ Therefore, neck management plays a key role in overall therapeutic planning.

In the last decades, the discussion for the patients with no evidence of lymph node disease was polarized between a selective neck dissection homolateral to the primary lesion^10^ and the expectant management with strict observation of the patient’s evolution (watchful waiting), reserving neck dissection only for those who develop metastatic lymph node disease over time.^11^, ^12^ Elective neck dissection supporters base their arguments on apparent better oncologic outcomes,^9^, ^13^ while those who advocates the watchful waiting draw attention to possible secondary sequels to neck dissection.^14^, ^15^, ^16^ The defenders of this conservative approach are based on the fact that approximately 75% of the patients do not have evidence of lymph node involvement after anatomopathological analysis of elective neck dissection.^5^

Sentinel Lymph Node Biopsy (SLB) appears as an alternative option apparently satisfactory from the oncological point of view and with less morbidity than selective neck dissection, since its main proposal is the identification and surgical removal of the first drainage lymph nodes from the site affected by the disease through a minimal and directed neck dissection.^17^, ^18^ Thus, most clinically N0 patients can be spared from neck dissection, which is only for the regional staging of pN0 patients. Thus, the SLB opens the perspective of obtaining a lower impact on the quality of life in this group of patients, without compromising oncological radicality.

This study aims to describe the initial oncological results of a prospective study of a Brazilian cancer reference center using Sentinel Lymph Node Biopsy (SLB) as part of the surgical treatment of squamous cell carcinoma T1/T2N0 of the oral cavity in comparison to the results of other patients with tumors of oral cavity of the same histological type and staging submitted to Selective Neck Dissection of levels I, II and III (SND) in the same institution.

## Methods

### Study design

This is a prospective, non-randomized, quasi-experimental study^19^ in which patients with early tumors of the mouth and with clinically negative neck (T1/T2N0) underwent neck surgical treatment through Sentinel Lymph Node Biopsy (SLB). The exclusion criteria for SLB were prior of treatment of other malignant tumors in the head and neck region; anterior cervicotomy in the lymph node drainage regions of the oral cavity; tumors whose transoral resection would not be possible in a combined cervical approach. In the control group, a series of patients with carcinomas of the mouth with the same staging that were submitted to Selective Neck Dissection of levels I, II and III (SND) treated at the same institution and in the same period were included. This group included patients whose tumors were not possible for a safe resection and those who did not accepted SLB.

Enty patients admitted between January 2012 and January 2015 in the Head and Neck Surgery Service of the Instituto do Câncer do Estado de São Paulo were included in the study. The project was approved by the Research Ethics Committee of the Institution under number 686/14 and informed consent form was applied to the participants, after an explanation in accessible language, patients with early-onset tumors (cT1 and cT2) with clinically negative neck (cN0), without any previous or treatment of other tumors in the head and neck territory were included in the study.

Clinical characteristics such as gender, age, primary tumor site was collected. In addition to the histological type, Pathological staging (pTNM), presence of positive margins, tumor thickness, presence of perineural invasion and angiolymphatic invasion, capsular spread in cases of positive lymph node metastases. Data were also collected on the need and types of adjuvant treatments performed.

### Sentinel lymph node method

The technique adopted combined planar lymphoscintigraphy with SPECT-CT. The radiopharmaceutical used was 99mTc-Dextran-70. Injections were performed under the direct visualization of the tumor, ranging from 2 to 4 peritumoral injections depending on the size or the accessibility of the lesion. The exams were performed in a Symbian-16/SIEMENS SPECT-CT hybrid device. When possible, sequential dynamic flat images were performed immediately after the injection of the radiopharmaceutical. All cases performed static flat images on the anterior, posterior and lateral projections of the head and neck.

After the analysis of flat and tomographic images by a nuclear medicine specialist, the capillary lymph nodes were identified according to number, radiopharmaceutical concentration, tomographic pattern (size, shape, density) and anatomical location according to the chain and cervical level. Cutaneous demarcation was performed in the projection of sentinel lymph nodes.

In the operative procedure, a gamma-probe was used to identify lymph nodes with detection ‒ considered as sentinels ‒ in the topography previously demarcated. Ex vivo detection with gamma probe was always performed to confirm detection in vivo. Confirmed as a sentinel lymph node, the detection count was recorded in 10 s ex vivo with the gamma probe. Excised lymph nodes without ex vivo detection was termed para-sentinels. After excision of the lymph nodes identified in the SPECT-CT and any other lymph nodes captured by the gamma probe intraoperatively, the surgical bed was scanned with the gamma probe with a measurement of the number of counts in 10 s. The procedure was considered as finished when the number of counts within 10 s of the operative bed was less than 10% the number of counts within 10 s of the highest capture sentinel node.

All sentinel lymph nodes removed were submitted to anatomopathological study with serial cuts of 3 μm, stained with hematoxylin and eosin. In doubtful cases, additional immunohistochemical examination for A1‒A3 cytokeratins was also performed. Patients with sentinel lymph nodes compromised by neoplasia underwent modified radical neck dissection within four weeks of the first procedure.

### Outcomes

The primary endpoint was disease-free survival compared between the two groups. As secondary outcomes, we considered overall survival, incidence of regional relapse, the incidence of the second primary tumor, and incidence of distant metastasis.

### Statistical analysis

SPSS version 17 (SPSS Inc.®, Chicago, USA) was used for statistical analysis. Mean and standard deviation calculations were used for continuous quantitative variables. For the qualitative variables, the frequency distributions were tabulated with the Chi-Square test. The Student’s *t*-test was applied in the comparisons of the quantitative variables after determination of parametricity by the Kolmogorov-Smirnov test. The Log- Rank test and Kaplan-Meier curves were used for the survival analyses. The efficacy of SLB as a method of diagnosis of occult metastases through the Receiver Operating Characteristic (ROC) curve with 95% Confidence Intervals (95% CI) was also evaluated; *p* < 0.05 was considered a statistically significant association.

## Results

A total of 70 patients were eligible for the study, 35 of them for each group. The age at SND ranged from 39 to 93 years old with a mean of 61.9 years old and a standard deviation of 11.4 years old, while in the SLB, it ranged from 39 to 83 years old with a mean of 59.8 years old and a standard deviation of 10.4 years old. The gender distribution was 28 men (80%) and 7 women (20%) in the SND; and 24 men (68.6%) and 11 women (31.4%) in the SLB.

In the SND, the most common primary site was the tongue with 11 patients (31.4%), followed by the lip with 9 (25.7%) and the retromolar area with 7 (20.0%). In the SLB, oral tongue was the most common primary site with 24 patients (68.6%), followed by floor of the mouth with 9 (25.7%) and buccal mucosa with 2 (5.7%). The clinical characteristics of the patients are summarized in Table 1. All patients had confirmed squamous cell carcinoma in the definitive pathology examination. Free margins were obtained in all resections.

**Table 1:** Descriptive data of patients submitted to Selective Neck Dissection (SND) and Sentinel Lymph Node Biopsy (SLB) and analysis of group homogeneity.

Variables	SND	SLB	*p*^a^
*Demographic data*			
Male gender	28 (80.0%)	24 (68.6%)	0.274
Age (years old)	b59.8 ± 10.4	c61.9 ± 11.4	0.400
*Subsidiary of primary tumor*			
Retromolar area	7 (20.0%)	‒	0.010
Lip	9 (25.7%)	‒	
Tongue	11 (31.4%)	24 (68.6%)	
Hard palate	1 (2.9%)	–	
Floor of the mouth	6 (17.1%)	9 (25.7%)	
Buccal mucosa	1 (2.9%)	2 (5.7%)	
*Characteristics of tumor*			
Perineural invasion	10 (28.6%)	1 (2.9%)	0.030
Angiolymphatic invasion	2 (5.7%)	0 (0.0%)	0.150
Tumor thickness (cm)	d1.1 ± 0.8	e0.8 ± 0.5	0.600
*PT Stages*			
pT1	13 (37.1%)	27 (77.1%)	0.001
pT2	22 (62.9%)	6 (17.1%)	
pT3	–	2 (5.7%)	
Lymph node metastases	6 (17.1%)	7 (20.0%)	0.760
Extracapsular spread	4 (66.7%)	2 (28.6%)	0.730
*Final stage*			
I	12 (34.3%)	24 (68.6%)	0.020
II	17 (48.6%)	3 (8.6%)	
III	2 (5.7%)	4 (11.4%)	
IV	4 (11.4%)	4 (11.4%)	
*Follow-up*			
Adjuvant radiotherapy	10 (28.6%)	8 (22.9%)	0.580
Concomitant chemotherapy	2 (5.7%)	3 (8.6%)	0.640
Locoregional relapse	6 (17.1%)	7 (20.0%)	‒
Distant Metastases	1 (2.9%)	2 (5.7%)	‒
Second primary tumor	3 (8.8%)	2 (5.7%)	‒
Deaths	6 (17.1%)	10 (28.6%)	‒
Follow-up time (months)	22.5 ± 16.7	28.9 ± 15.6	‒

a Chi-Square test applied in the comparisons between qualitative variables and Student’s *t*-test in the other analyses.

b Mean ± standard deviation (range 39–93 years old).

c Mean ± standard deviation (range: 39–83 years old).

d Mean ± standard deviation (range: 0.1–3.9 cm).

e Mean ± standard deviation (range: 0.2–2.0 cm).

Under the homogeneity test (Table 1), the groups were balanced for distribution by gender (*p* = 0.270), age (*p* = 0.400), free margins, angiolymphatic invasion (*p* = 0.150), tumor thickness (*p* = 0.60), number of lymph nodes involved (*p* = 0.570), neck status pN (0.760), pN staging (0.540), extracapsular spread of lymph node metastases (*p* = 0.730), adjuvant radiotherapy (*p* = 580) and chemotherapy (0.640). However, we observed an imbalance in the criteria of primary site (*p* = 0.010), perineural invasion (*p* = 0.030), and anatomicopathological evaluation of T (*p* = 0.001) in early stages T1 and T2 (*p* = 0.020).

In SND, 13 (37.1%) of the patients had tumors classified as T1 and 22 as T2 (62.9%). The tumor thickness ranged from 0.1 to 3.9 cm with the mean of 1.1 cm and the standard deviation of 0.8 cm. Angiolymphatic invasion was detected in 2 patients (5.7%) and perineural invasion in 10 patients (28.6%). Occult metastases were found in cervical lymph nodes in 17.1% (6 patients). Of the patients with compromised lymph nodes, the incidence of extracapsular spread was observed in 66.7% (4 patients). In total, 82.9% of the cases were considered as stages I and II.

In the SLB group, 27 patients had tumors classified as pT1 (77.1%), 6 patients as pT2 (17.1%) and 2 patients as pT3 (5.7%; these two patients were clinically staged as T2 in the preoperative period; definitive anatomopathological examination were reclassified as pT3). Tumor thickness ranged from 0.2 to 2.0 cm with a mean of 0.8 cm and a standard deviation of 0.5 cm. Only one patient (2.9%) had perineural invasion, while no angiolymphatic invasion was detected in any patient. Occult metastases in the lymph nodes of this group were observed in 7 patients (20%). Of them, the incidence of extracapsular spread was 28.6% (2 patients) and 77.1% of the cases were classified as stages I and II.

Regarding the adjuvant treatment, 10 patients (28.6%) received radiotherapy and 2 (5.7%) had concomitant chemotherapy in the SND. While in SLB, 8 patients (22.9%) and 3 (8.3%) had the indication of radiotherapy and chemotherapy, respectively.

For SND, follow-up ranged from 2 to 57 months, with a mean of 22.5 and a standard deviation of 16.7 months. Locoregional recurrence occurred in 17.1% of the cases (6 patients) with a 2.9% development of distant metastasis (1 patient) and 8.8% developed a second primary tumor. At BSL, follow-up ranged from 3 to 61 months, with a mean of 28.9 and a standard deviation of 15.6. Locoregional recurrence was observed in 20.0% (7 patients), 5.7% had distant metastasis (2 patients, one without locoregional recurrence) and 2 patients (5.7%) had a second primary tumor.

**Fig. 1:**
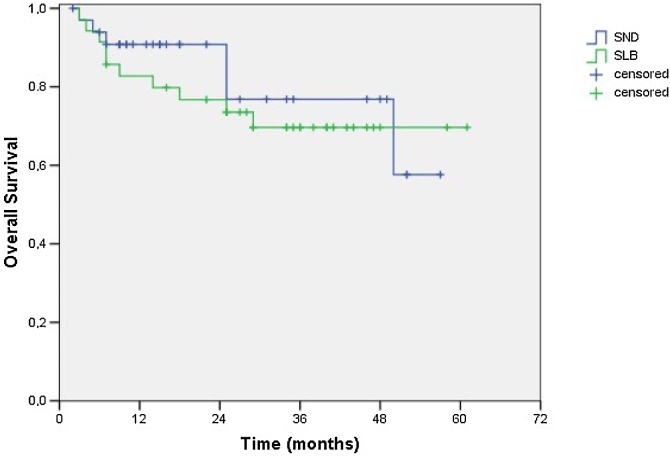
Cumulative overall survival of 57.6% for the SND group and 69.7% for the SLB (*p* = 0.521 ‒ Log-Rank test).

**Fig. 2:**
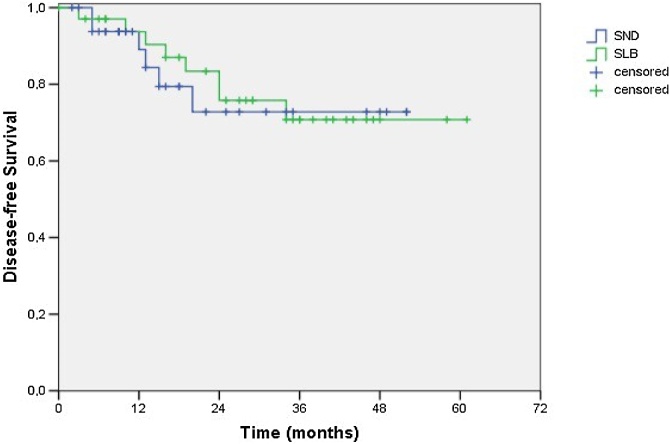
Cumulative disease-free survival of 73.0% for the SND group and 71.5% for the SLB group (*p* = 0.753 ‒ Log-Rank test).

During the follow-up period, there were 6 deaths in SND; 4 of these deaths were related to neoplastic disease. In SLB, we observed 10 deaths, 5 of them related to the tumor disease. Overall survival was 57.6% for SND and 69.7% for SLB (*p* = 0.521 ‒ Log-Rank test; Fig. 1). Disease-free survival was 73.0% for the SND and 71.5% for the SLB (p = 0.753 ‒ Log-Rank test; Fig. 2).

**Fig. 3:**
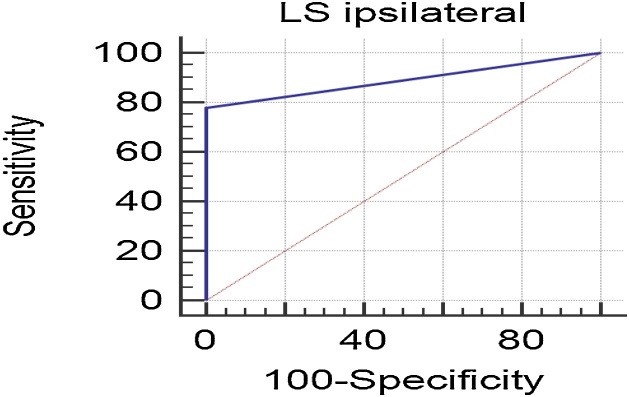
ROC curve for the diagnostic evaluation of sentinel lymph node biopsy in patients with squamous cell carcinoma of the oral cavity. Area under the ROC curve = 0.889 (95% CI 0.734%‒0.970%).

The accuracy of the sentinel lymph node biopsy to detect occult metastases was 88.9% (95% CI 73.4%‒97.0%, Fig. 3), with just one case of false-negative result. The sensitivity was of 77.8% (95% CI 40.0%‒97.2%), the specificity and also the negative predictive value was of 100.0 (95% CI 86.3%‒100.0%), and the positive predictive value was of 92.6% (95% CI 81.7%‒99.9%).

## Discussion

Neck dissection operation had a positive impact on the survival of innumerable patients with cancers in the head and neck region for almost a century.^10^, ^20^ It was so iconic that it became the symbolic operation of Head and Neck Surgery.^21^, ^22^ However, since its initial description in English literature in 1906 by George W. Crile,^23^, ^24^ proposals for modifications have arisen, derived from the original operation, called classical radical neck dissection. These changes were aimed at restricting the extent of resection of both lymphatic and non-lymphatic structures, aiming to reduce the mortality associated with the procedure.^25^, ^26^

In the case of patients with early tumors of the oral cavity without clinical evidence of lymph node metastases can occur in 20% to 30% of cases, the amount of patients who would not benefit from elective neck dissection would correspond to more than two thirds of the cases, and still with the risk of suffering the complications of the procedure.^5^, ^8^ The SLB has the requisites to fill this gap, since its intention is to promote the surgical removal of only the lymph nodes really at risk of disease, saving the patient from wider, possibly unnecessary dissections, minimizing the risk of associated complications.^15^, ^27^

The concept that a lymph node or a restricted group of lymph nodes is initially affected by micro-metastases, before a more extensive lymph node spread of the disease, was theorized by Cabañas in 1977 for penile carcinoma.^28^ This lymph node or this group of lymph nodes was named sentinel lymph node(s). Later, this theory was applied to melanomas and detailed by Morton.^29^, ^30^ With the observed success in melanoma and breast cancer, SLB has been accredited to be used in the treatment of head and neck carcinomas.^31^, ^32^ In the specific radiopharmaceutical case of the oral cavity, due to the easy access for the injection of the drug radio in the primary tumor, this site acquires status as an ideal candidate for the application of the method.^33^, ^34^, ^35^ Several studies have been published on adaptations and validations of intraoperative lymph node mapping techniques.^17^, ^36^ In 2001, the First International Conference on Sentinel Lymph Nodes applied to Head and Neck Surgery was held in Glasgow, United Kingdom.^37^ The results obtained in the several participating centers showed that the method has the same sensitivity for staging of clinically negative necks as compared to selective neck dissection. In 2016, the 7th Conference was held in the city of Rome, with validation of these results through robust case studies from several centers, mainly from Europe and proposals for new methodologies to increase the accuracy of the method with the use of new radiopharmaceuticals, fluorescein and radioguided navigation.^38^

In our institution, implantation of the method was possible by the aggregation of referral services in Head and Neck Surgery, Pathology and Nuclear Medicine. Nevertheless, due to the relatively small number of early mouth tumors, we chose to adopt the almost experimental prospective study design, which is very commonly used in the evaluation of surgical procedures; due to the lack of practicality in masking or randomizing the control and intervention groups.^39^, ^40^

The association of SPECT-CT with lymphoscintigraphy is relatively recent. The technique was used in patients with oral tumors in the study of Khafif et al. in 2006.^41^ In the surgical cohort described by Haerle et al., the authors had the strong impression that the procedures were facilitated and abbreviated with the preoperative topographic orientation of the sentinel lymph nodes.^42^ We share the same impression to explain the results obtained. Objectively, we had a perfect match between the levels and the sides of the SPECT and gamma probe in 100% of cases.

In our study, SLB was very reliable as a diagnostic method for the detection of micro-metastases, with high sensitivity and specificity (77.8% and 100.0%, respectively), and respectable predictive values: positive of 92.6% and negative of 100.0%. Results are in accordance with the researched literature.^42^, ^43^, ^44^, ^45^

In the cases that evolved to death, we had four patients who presented second primary tumors (two of the esophagus, one of the pancreases and one of the colon). In the SLB group, two pNs0 patients presented cervical recurrence contralateral to the sentinel lymph node; one during of radiotherapy, in a rapidly progressive way, without time to perform a salvage surgery; another was submitted to a large salvage operation including mandibulectomy, and he is currently under clinical follow-up, with no evidence of disease at the last visit. Only one patient in the SLB pNs0 group presented a homolateral recurrence, characterizing a false negative case. Such patient was submitted to a salvage neck dissection but ended up evolving to death by the disease.

The similar results obtained between the SLB and SND groups in the overall survival and disease-free survival, associated with the reduced number of false-negative cases observed to date, credit SLB as standard behavior in the near future in services with necessary conditions for the reproducibility of the method. As a most important condition for the success of the method, we see the good integration between head and neck sugery, pathology and nuclear medicine services, pathological anatomy and nuclear medicine. Although SPECT-CT has proved to be of great value in our experience, it should not be considered as an indispensable condition for the implantation of the method. The planar lymphoscintigraphy can guide the surgeon of the position of the capturing lymph nodes, especially their laterality. Adequate use of the intraoperative gamma probe is a key element in the identification of the sentinel node(s) and should not be replaced, not even associated with patent blue injection, a procedure abandoned by almost all great experience.^35^

The intraoperative sentinel lymph node frozen session, in an attempt to avoid the second procedure in pNs+ patients is controversial and is not used by most services, due to the technical limitations of this method in the identification of micro-metastases and the risk of loss of material for an adequate histopathological examination.^35^

Our work contributes to the literature since it proves, through an original prospective, non-randomized, quasi-experimental study, that SLB is comparable to SND with regard to the results of oncological treatment and the accuracy of detection of micro-metastases in patients with epidermoid carcinoma of the oral cavity squamous cell carcinoma cT1/T2N0. There is no published literature similar to the research presented here. The criticism of our work is the disproportion between cases pT1 and pT2 between the two groups, with preponderance of the former in the SLB group, unlike the SND group where pT2 cases predominated. This discrepancy is explained by the fact that many T2 cases were directed to the SND group because, due to the thickness of the primary tumor, an oncologically safe transoral resection was not possible, requiring a combined oro-cervical approach, exclusion condition to the SLB in our service.

We believe that, although evidence-based medicine would require randomized controlled clinical studies, comparing conventional and alternative treatments, for example using non-inferiority screening,^46^ we questioned whether study would be feasible, even at multi-institutional level, since the number of patients needed in each arm would be very high, associated with the lack of early cases eligible for the method. The ethical discussion in randomizing this group of patients is associated with this fact, offering for them a treatment with greater morbidity and similar oncological results, as already demonstrated in the literature and the results presented here.

## Conclusion

Our study has shown that the SPECT-CT-assisted sentinel lymph node biopsy technique is a reliable method for the treatment of clinically negative necks in early oral cavity tumors.

## Financial support

None.

## Declaration of competing interest

The authors declare no conflicts of interest.
